# Meeting the needs of children with congenital and developmental cataract in Africa

**Published:** 2008-03

**Authors:** Paul Courtright, Childhood Cataract Experts Meeting Group

**Affiliations:** Kilimanjaro Centre for Community Ophthalmology PO Box 2254, Moshi, Tanzania

**Figure:**
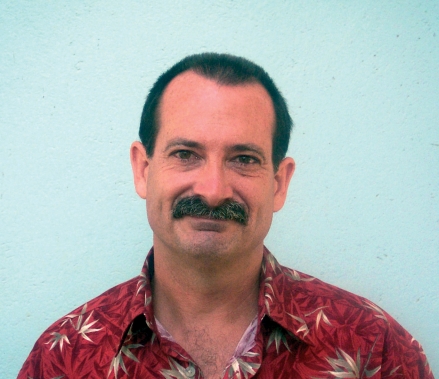


In much of Africa, childhood cataract is becoming one of the leading causes of new cases of blindness reported per year.^1^

Although there is insufficient data on childhood cataract, both congenital and developmental, the backlog of children in need of surgery is estimated to be around 100 children per million population. The number of new cases of corneal blindness is estimated to be around 20 children per million population per year.^2^^,^^3^

The World Health Organization (WHO) recommends that there be one paediatric ophthalmology tertiary centre per 10 million population^4^; however, few countries in Africa have reached this target. Even in settings with tertiary centres, few children are brought in for surgery; of those who are, most are brought in too late for surgeons to be able to achieve outcomes of the highest quality.^5^ This can be explained by the fact that both communities and health care providers are not sufficiently aware that children can develop cataract. The lack of programmes to identify and refer children with cataract compounds this problem. It also needs to be noted that, in most hospitals or other surgical facilities, boys outnumber girls; this is primarily due to cultural constraints and does not reflect any recognised biological risk factors associated with males.^5^^,^^6^

Virtually all children receiving surgery for congenital cataract in Africa will require long term follow-up for refractive error correction and low vision care. However, follow-up remains either patchy or non-existent.^7^

**Figure:**
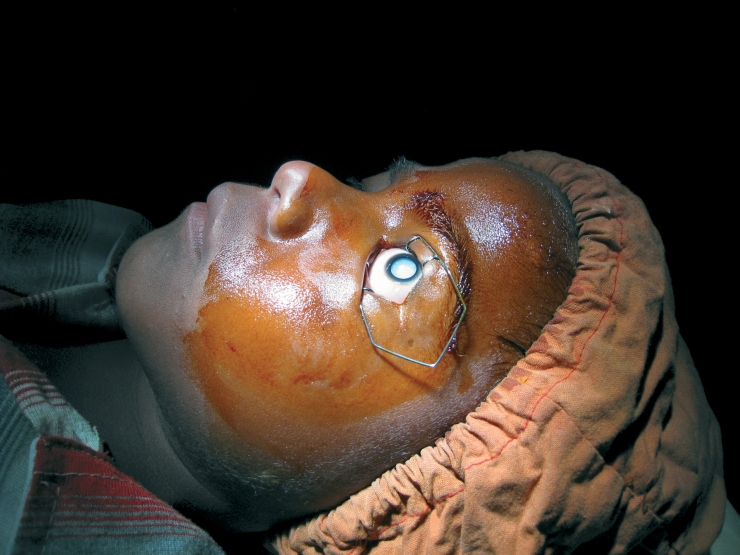
**A child prepared for cataract surgery. NEPAL**

Although they are still few in number, tertiary facilities for paediatric ophthalmology in Africa have strengthened the quality of their surgical services over the past three to five years. Unfortunately, better surgical services have not usually been matched by improvement in the other services needed to strengthen overall management of congenital and developmental cataract. These include better programmes promoting early identification and referral, adequate follow-up, provision of spectacles, low vision care, or referral to inclusive education.

In May 2007, the Kilimanjaro Centre for Community Ophthalmology (KCCO), with financial support from Dark & Light Blind Care, brought together 18 people from throughout Africa, as well as key personnel from Europe and Asia, to draft recommendations and to prepare a practical manual of best practice for the management of childhood cataract in Africa. This manual will be available by August 2008. Materials from the workshop are available on the KCCO website: www.kcco.net

## Recommendations

### Efforts needed at the national level in Africa to address childhood cataract

Throughout Africa, national prevention of blindness or VISION 2020 committees need to identify existing tertiary centres for paediatric ophthalmology. In collaboration with these centres, they need to define each centre's respective catchment area (population: 10 million). For each catchment area, existing data on childhood cataract should be compiled (including age, sex, and place of residence). For planning and monitoring purposes, a childhood cataract surgical rate should be calculated for each VISION 2020 district (population: one to two million). The childhood cataract surgical rate states how many cataract operations should be performed in each district per year in order to eliminate the condition among the children residing in the district.

This information should be used to identify districts where the actual number of cataract operations performed is too low and to monitor annual progress towards reducing childhood blindness due to cataract.

### Identifying and referring children with cataract

Evidence suggests that the use of key informants (at the community level) may increase identification and referral of children requiring surgery, particularly when local cultures are relatively cohesive and when people living in the geographic area know each other.^8^^,9^ Additional research is needed to test this method for sustainability and to test it in other settings. It should also be compared with other approaches, such as the use of health workers (for example, those doing immunisations).

In many countries, children with cataract are still admitted to schools for the blind. National policies on admission of children to schools for the blind (in particular, ophthalmological examination prior to admission) are either absent or not enforced. All those engaged in eye care, education, and rehabilitation of children need to collaborate to first provide children with the best possible eye care before placing them in appropriate educational settings.

The exact timing of cataract surgery depends upon the individual characteristics of each child. However, when a child's pupil is white, health care staff must treat this as an emergency and ensure that the child is seen by an ophthalmologist as soon as possible. To ensure this, the curricula for training mid-level and primary level health providers should be reviewed and upgraded, if necessary. It would also be helpful to improve the knowledge and skills of existing care providers.

### Financing cataract surgery in children

For a paediatric ophthalmology tertiary centre, the cost of equipment, consumables (e.g. high-power intraocular lenses and spectacle frames for babies and small children), and recurrent expenses are very high and require adequate funding. In most cases where programmes have achieved a significant increase in the number of operations in children, it has been necessary to waive surgical fees and to reimburse much of the travel expenses incurred by families bringing children for surgery and follow-up. These approaches may need to be considered in most settings in Africa and will require dedicated financial support.

**Figure:**
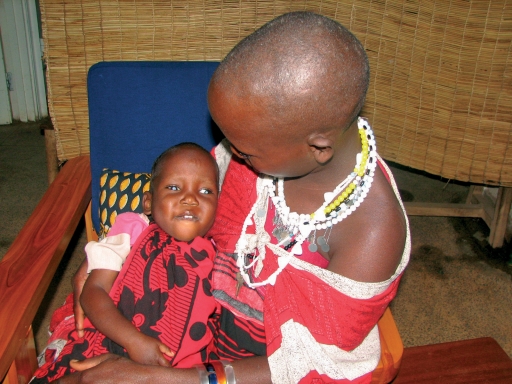
**A girl with bilateral cataract. TANZANIA**

### Surgical intervention and surgical facilities

All children (in particular, younger children) should only be operated on by paediatric ophthalmologists in well-equipped tertiary centres; these centres need to ensure that high-quality paediatric anaesthetic services are available. A paediatric ophthalmology tertiary centre should have a ‘childhood blindness coordinator’ on the staff. This person will be responsible for counselling parents (and older children), organising activities for early detection, training health staff, and implementing a tracking system to ensure that children are brought back for follow-up, spectacles, and low vision care. Every tertiary centre should have facilities to provide low vision services and spectacles for children.

Follow-up after surgery is essential throughout childhood. All centres providing surgery should adopt strategies that have been shown to be effective in promoting regular follow-up; these strategies include counselling, keeping good records, using cell phones (mobile phones) to keep in touch, reimbursing transport costs, and organising family visits by local eye care workers.

### Decentralisation of follow-up services

Low vision services and some refractive services should be decentralised as much as possible, in particular because refractive error changes periodically in children. Relevant personnel in districts served by the tertiary centre should be trained to provide basic follow-up services. It will be challenging to link tertiary and district services to ensure that children are kept track of. To ensure good practice, it is essential to establish systems of accountability.

### Linking eye care, low vision, education, and rehabilitation services

Successful low vision service provision requires strong links between eye care and low vision services, as well as accurate refraction and near vision assessment. In addition, low vision services should have links with education and rehabilitation services. In most settings, special education teachers and rehabilitation workers need additional training. Eye care providers need to take the lead in initiating and maintaining low vision and educational support of children.
